# Diaphragmatic Hernia Repair Using Biosynthetic Tissue Reinforcement Patch: A Case Series Experience

**DOI:** 10.7759/cureus.4274

**Published:** 2019-03-19

**Authors:** David Rubay, Levonti Ohanisian, StevenClaude D White, Mohammed Al-Musawi

**Affiliations:** 1 Surgery, Florida Atlantic University Charles E. Schmidt College of Medicine, Boca Raton, USA; 2 Internal Medicine, Florida Atlantic University Charles E. Schmidt College of Medicine, Boca Raton, USA; 3 Genetics, Emory University, Atlanta, USA; 4 Surgery, Anschutz Medical Campus, University of Colorado, Aurora, USA

**Keywords:** diaphragmatic hernia, tissue reinforcement

## Abstract

Diaphragmatic hernias are commonly encountered by general surgeons. However, repair is often fraught with complications and recurrence. The use of extracellular matrix scaffolds for repair of damaged tissues through constructive remodeling is an effective surgical adjunct. Herein, we describe the repair of diaphragmatic hernias using GORE® BIO-A® Tissue Reinforcement patch in a series of patients.

## Introduction

Diaphragmatic hernias are commonly encountered by general surgeons. The hernia defect is typically repaired surgically, however, there are minor variations in technique. Based on defect size, location, margins, and risk of infection, there are different recommendations with regards to the preferred approach and repair material [1]. One particular variable is the risk of infection of the hernia repair which can be confounded by the use of synthetic non-absorbable materials. In this case series, we share our experiences with the use of GORE® BIO-A® Tissue Reinforcement (W. L. Gore & Associates, Flagstaff, AZ) to repair and reinforce the closure of a diaphragmatic hernia in three patients who were considered to have large defects which required patch closure. The goal was to use a material which would enhance tissue growth and minimize future risk of infection associated with use of permanent synthetic material. We were able to reduce the hernia and strengthen the diaphragmatic muscle through the incorporation of GORE® BIO-A® Tissue Reinforcement, allowing for the proliferation of epithelial and progenitor cells along with reconstruction of the native tissue. GORE® BIO-A® Tissue Reinforcement is a biocompatible synthetic polymer that is neither from human nor animal tissue. It provides a 3D scaffold that allows a patient’s own collagen the opportunity to grow over and through it while the main scaffold is gradually absorbed [2].

## Case presentation

Patient #1

The patient is a 70-year-old male with type-2 diabetes mellitus (DM) and chronic kidney disease (CKD) with a history of chronic abdominal discomfort who presented with a missed posterior congenital right Bochdalek hernia. Abdominal computed tomography (CT) revealed a posterior right diaphragmatic hernia containing loops of non-incarcerated bowel without obstruction. The operation was performed via a midline laparotomy and bowel loops were pulled from the hernia inadvertently creating a small enterotomy which was repaired primarily. A pleural sac covering the contents was identified but not opened. The hernia orifice was 8 x 6 cm with a muscular posterior rim. After reducing the orifice to 4 x 4 cm using sutures at the angles, the GORE® BIO-A® Tissue Reinforcement patch was used to close the defect without tension using multiple interrupted nonabsorbable 4/0 prolene sutures. The patient’s associated comorbidities and the enterotomy that was encountered increased the likelihood of surgical infection which led us to use the aforementioned technique and tissue reinforcement patch.

Patient #2

The patient is a 6-year-old male with no significant past medical history who was admitted with a strangulated Morgagni hernia, sepsis and reactionary pericardial effusion. A midline laparotomy was performed and bowel was pulled out of the hernial sac. The ischemic small bowel was resected with the primary end to end anastomosis. The rim of the defect was identified and dissected circumferentially. The defect was repaired using a GORE® BIO-A® Tissue Reinforcement patch with the same technique as mentioned above. In the setting of an infected field, this patient was deemed to be at increased risk of infection and therefore a good candidate for GORE® BIO-A® Tissue Reinforcement patch.

Patient #3

The patient is a 15-year-old male who presented with the recurrence of a posterior left Bochdalek hernia previously repaired ten years ago via laparotomy. The previous repair was done by direct suturing without patch placement. A left posterior lateral thoracotomy was done to avoid abdominal adhesions. The bowel was released from lung and pleura and the hernia rim was dissected and identified. After reducing bowel, two serosal tears were identified which were closed primarily. The 6 x 5-cm hernia was reduced to 3 x 3 cm without tension and the residual defect was closed using GORE® BIO-A® Tissue Reinforcement patch using the same technique as mentioned above. Multiple operations, which are associated with bowel perforation, placed this patient at high risk for surgical infection.

## Discussion

**Figure 1:**
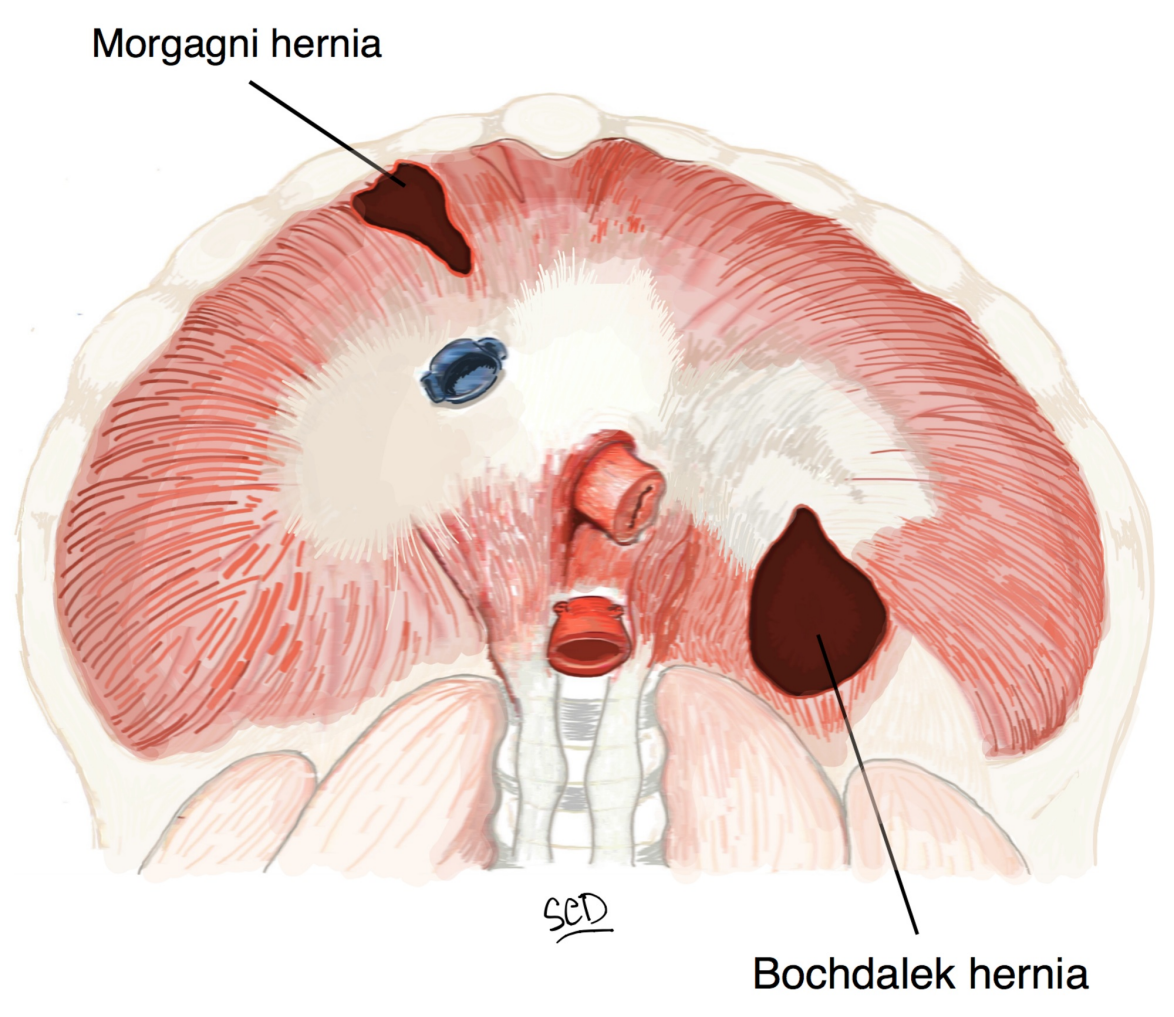
Illustration depicting Bochdalek and Morgagni hernia defects

Diaphragmatic hernias are common and approximately 350,000 repairs are completed annually with a little more than half performed in an outpatient setting [2]. Estimates of surgical site infection in open repairs range from 3-14% [3-4]. The patients in our case series had either Bochdalek or Morgagni hernias (Figure 1) and were all at increased risk for infection [5].

**Figure 2:**
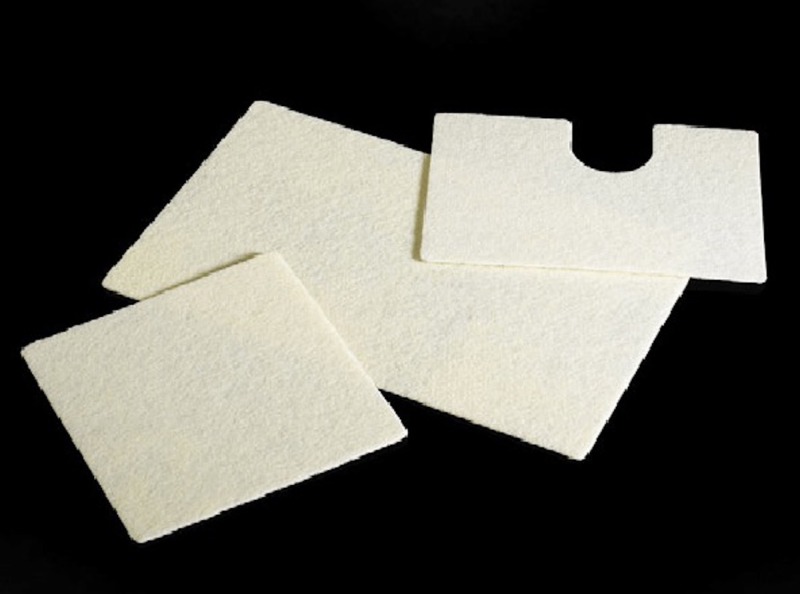
Gore Bio-A tissue reinforcement

As in any hernia repair, the goal for each patient was to achieve tension-free closure. The use of mesh has become standard practice in achieving such closure although usage rates between synthetic and biological mesh products are varied. As recommended by the Diaphragmatic Hernia Working Group, in patients with an elevated risk of infection, biologics are the repair material of choice due to increased resistance to infection and because biologics do not necessitate removal in the setting of infection [1]. Considering the risks of infection in our cases above, the decision was made to use an implant with enough strength to hold the repair and to work as a scaffold for the patient’s own tissue to fill the defect and later become gradually absorbed. GORE® BIO-A® Tissue Reinforcement patch has been shown in several studies to be a promising material that promotes vascularization, regeneration, and tissue growth while providing strong structural support in inguinal hernia and hiatal hernia repairs [6-9] (Figure 2).

**Figure 3:**
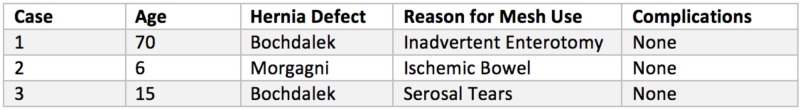
Summary table of case series

Another reason why we chose to use this tissue reinforcement patch was it has been used successfully in the case of a dehisced abdominal wound [10]. The patch’s regeneration capability has been hypothesized to be the primary mechanism resulting in reduced infections [11]. We found the material was easy to use and we did not encounter difficulties in suturing the material to the diaphragmatic muscle. Also, in the follow-up imaging, we performed on our patients ensured the material maintained adequate durability. Below, we summarize the case series discussed (Figure 3).

## Conclusions

Diaphragmatic hernia repairs are a commonly performed surgery. Certain risk factors, such as increased risk of infection, may necessitate alterations of the standard approach to the operation. We were able to achieve positive results by using a GORE® BIO-A® Tissue Reinforcement patch to repair diaphragmatic defects in patients who were at high risk for infection was effective in our experience due to the patch’s capability to rely on collagen regeneration within the patch scaffolding as well as the absorptive qualities of the semisynthetic patch itself without leaving any foreign body in place. Further follow-up and imaging studies are needed to determine long term outcomes and assimilation of the GORE® BIO-A® Tissue Reinforcement patch in the native diaphragmatic tissue. Early results using GORE® BIO-A® Tissue Reinforcement patch are promising and the use of this patch has shown regeneration in several different tissues along with strong structural support.
